# Adeno-associated virus vectors for retinal gene therapy in basic research and clinical studies

**DOI:** 10.3389/fmed.2023.1310050

**Published:** 2023-12-01

**Authors:** Xue Xia, Xinzheng Guo

**Affiliations:** State Key Laboratory of Common Mechanism Research for Major Diseases, Suzhou Institute of Systems Medicine, Chinese Academy of Medical Sciences and Peking Union Medical College, Suzhou, China

**Keywords:** adeno-associated virus, vector, retina, gene therapy, glaucoma, age-related macular degeneration, diabetic retinopathy, inherited retinal diseases

## Abstract

Retinal degenerative diseases, including glaucoma, age-related macular degeneration, diabetic retinopathy, and a broad range of inherited retinal diseases, are leading causes of irreversible vision loss and blindness. Gene therapy is a promising and fast-growing strategy to treat both monogenic and multifactorial retinal disorders. Vectors for gene delivery are crucial for efficient and specific transfer of therapeutic gene(s) into target cells. AAV vectors are ideal for retinal gene therapy due to their inherent advantages in safety, gene expression stability, and amenability for directional engineering. The eye is a highly compartmentalized organ composed of multiple disease-related cell types. To determine a suitable AAV vector for a specific cell type, the route of administration and choice of AAV variant must be considered together. Here, we provide a brief overview of AAV vectors for gene transfer into important ocular cell types, including retinal pigment epithelium cells, photoreceptors, retinal ganglion cells, Müller glial cells, ciliary epithelial cells, trabecular meshwork cells, vascular endothelial cells, and pericytes, via distinct injection methods. By listing suitable AAV vectors in basic research and (pre)clinical studies, we aim to highlight the progress and unmet needs of AAV vectors in retinal gene therapy.

## 1 Introduction

**Figure 1:**
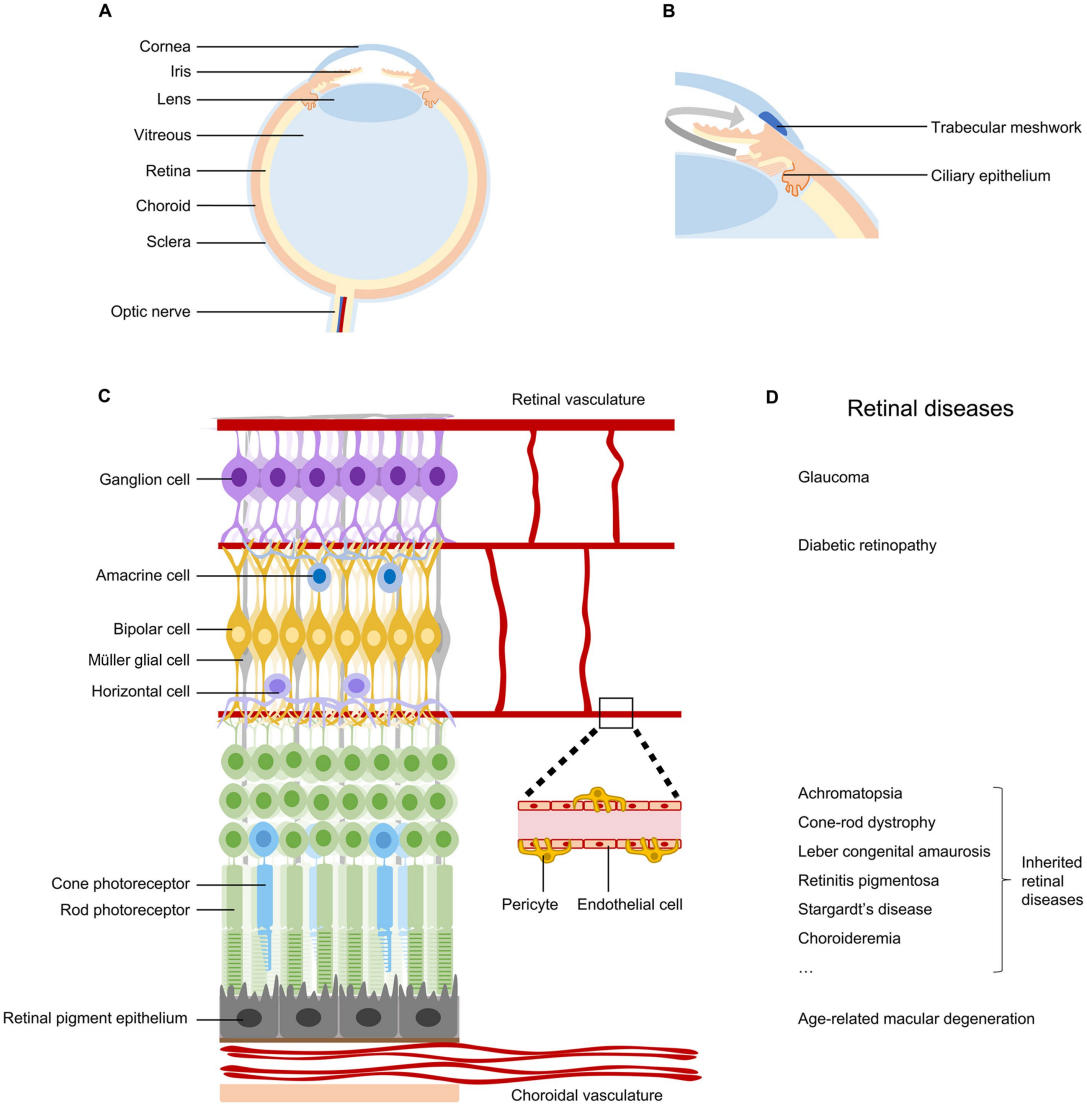
Anatomy and diseases of the retina. **(A)** Schematic diagram of the human eye, indicating cornea, iris, lens, vitreous, retina, choroid, sclera, and optic nerve. **(B)** Ocular tissues involved in the production and outflow of aqueous humor. **(C)** Anatomy of the retina, indicating retinal neurons, retinal pigment epithelial cells, Müller glial cells, endothelial cells, and pericytes in vascular systems. **(D)** Common retinal diseases that can lead to irreversible vision impairment.

The eye is the organ we use to sense light and form vision. As a sophisticated optical organ, it is composed of different tissues with distinct functions in a highly compartmentalized feature (1) (Figure 1A). In addition, the eye is filled with two types of humor, aqueous humor in the anterior segment and vitreous humor in the posterior segment of the eye, to keep its pressure and shape (2). The balance between the production and outflow of aqueous humor is important to maintain the eye’s normal intraocular pressure (IOP) (Figure 1B).

The retina, located at the back of the eyeball, is a layer of neural tissue that senses light and transmits visual information to the brain (Figure 1C). It consists of multiple types of neurons organized in three nuclear layers to produce a complex visual output. Photoreceptors (rods and cones) in the outer nuclear layer convert light into neural signals. Bipolar cells, horizontal cells, and amacrine cells in the inner nuclear layer modulate and relay visual signals. Retinal ganglion cells (RGCs) in the ganglion cell layer project their long axons and send visual information to brain targets for vision perception (3). The retina is nourished by two distinct vascular systems. The choroidal vasculature supplies oxygen and nutrients to the outer retina through the retinal pigment epithelium, while the retinal vasculature meets the metabolic requirements of the inner retina (4).

Numerous diseases can damage the retina and impair vision (Figure 1D). Conditions affecting the outflow of aqueous humor can lead to glaucoma, resulting in the degeneration of retinal ganglion cells and permanent vision loss (5). Problems with choroidal or retinal vascular structures are associated with age-related macular degeneration and diabetic retinopathy, respectively (4). Genetic mutations occurring in retinal pigment epithelium cells and photoreceptors can lead to a wide spectrum of inherited retinal diseases (6, 7). Currently, retinal diseases are the leading causes of irreversible vision loss and blindness. Recent global prevalence studies estimated that in 2020, there were 76 million patients with glaucoma (8), 196 million with age-related macular degeneration (9), 103 million with diabetic retinopathy (10), and 5.5 million with various inherited retinal diseases (11). With population aging and lifestyle changes, these significant vision-threatening diseases are expected to affect more people in the near future.

Nevertheless, the current treatment options for retinal diseases remain limited and inadequate. For example, for the management of glaucoma, the current mainstream therapy is to decrease IOP using topical eye drops. However, several challenges remain unresolved. First, anti-glaucomatous eye drops are associated with various local or systemic adverse effects, such as iris color change and cardiovascular problems (12). Second, it is difficult for some patients to control their IOP (13). Third, a significant proportion of glaucoma patients still progress to blindness despite receiving proper treatment to reduce IOP (14). Lastly, degeneration of RGCs is the direct cause of vision impairment in glaucoma, but protection of RGCs has not been widely adopted in clinical practice. It is imperative to develop new therapeutic strategies to confront the challenge of an increasing number of patients affected by various retinal diseases.

## 2 Retinal gene therapy

Gene therapy is a medical strategy that aims to treat or cure diseases by directly manipulating specific genes. Due to its easy accessibility and compartmentalized anatomy, the eye is a favorable organ for gene therapy (15, 16). In recent years, the genetic and pathogenetic mechanisms of many ocular diseases have been elucidated. In addition, effective gene manipulation techniques, such as gene editing, have been developed and widely employed. These advancements have made ocular gene therapy a fast-growing field in basic research and clinical studies.

Encouragingly, in 2017, the FDA approved the first gene therapy in the United States, Luxturna from Spark Therapeutics, Inc., to treat biallelic RPE65-mutation-associated retinal dystrophies that leads to vision loss (17), including some subtypes of retinitis pigmentosa and Leber congenital amaurosis (18). At present, there is a surge in retinal gene therapy clinical trials targeting a wide range of retinal diseases, including monogenic disorders such as Leber congenital amaurosis, retinitis pigmentosa, achromatopsia, choroideremia, X-linked retinoschisis, Leber hereditary optic neuropathy, and multifactorial disorders like wet age-related macular degeneration and diabetic retinopathy (16, 19–21).

Nevertheless, gene therapy research and clinical studies for the most prevalent retinal diseases, such as glaucoma, age-related macular degeneration, and diabetic retinopathy, remain very limited and cannot meet the needs of the vast and rapidly growing patient population worldwide.

## 3 AAV vectors for retinal gene therapy

Gene delivery vectors are critical for the efficient and specific transfer of therapeutic genes into target cells and thus for the efficacy and safety of gene therapy. Adeno-associated viruses (AAVs) are endowed with several key advantages suitable to be adopted as vectors for gene therapy. First, AAVs are replication-incompetent and non-pathogenic, have low immunogenicity, and exhibit low risk of insertional mutagenesis. These properties render AAV safe vectors for gene therapy (22). In addition, AAVs are capable of achieving stable and long-term expression of therapeutic genes in quiescent cells, such as neurons and myocytes, which is essential for the sustained efficacy of many gene therapy strategies (23). Furthermore, AAV capsid proteins, which encapsulate the AAV genome and determine its selection and entry of target cells, are highly versatile and amendable. This feature provides opportunities for AAV engineering to generate vectors with diverse tropisms for the treatment of a wide range of diseases (24, 25). Consequently, AAV vector stands as one of the leading platforms for gene therapy delivery.

**Figure 2:**
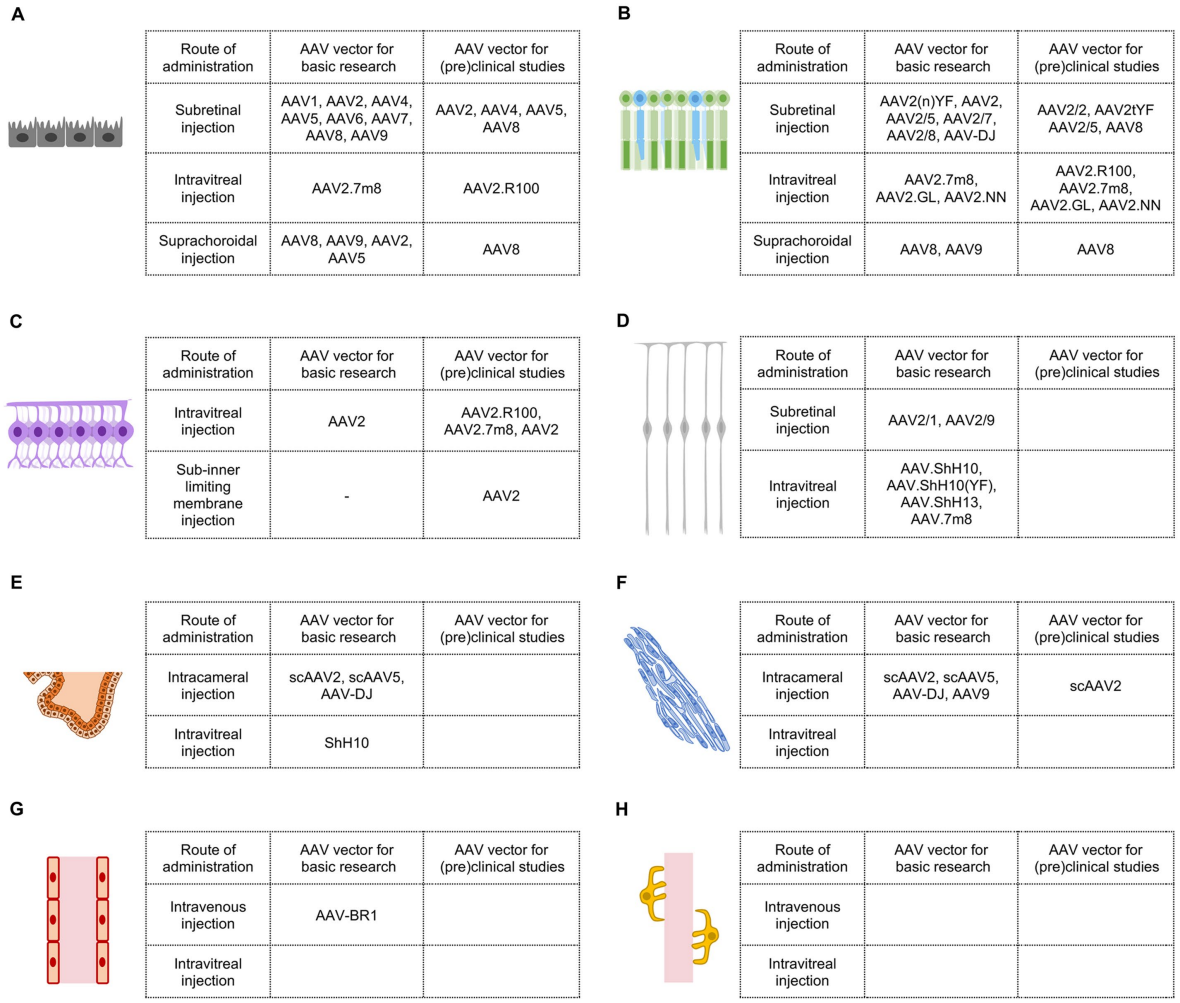
AAV vectors for retinal gene therapy in basic research and (pre)clinical studies. **(A)** Efficient AAV vectors targeting retinal pigment epithelial cells. **(B)** Efficient AAV vectors targeting photoreceptors. **(C)** Efficient AAV vectors targeting retinal ganglion cells. **(D)** Efficient AAV vectors targeting Müller glial cells. **(E)** Efficient AAV vectors targeting ciliary epithelial cells. **(F)** Efficient AAV vectors targeting trabecular meshwork cells. **(G)** Efficient AAV vectors targeting vascular endothelial cells in the eye. **(H)** Efficient AAV vectors targeting vascular pericytes in the eye.

In 1996, Ali et al. (26) first reported the evaluation of AAV as a gene transfer vector for the retina via subretinal injection and found AAV is capable of transducing retinal pigment epithelium and photoreceptors, supporting the use of AAV for gene therapy of retinal diseases such as retinitis pigmentosa. Since then, colleagues have made extensive efforts to discover or engineer improved AAV variants for ocular gene therapy (27, 28). The eye is a highly compartmentalized organ, consisting of multiple disease-related cell types. For efficient and specific gene transfer to target cell types, administration routes and AAV variants, two inextricably interwoven factors, must be carefully considered. In the following sections, we provide a brief overview of suitable AAV vectors and their corresponding administration routes for gene delivery into important ocular cell types, with a focus on studies conducted with rodents for progress in basic research and studies involving humans or non-human primates, which share the most similar eye anatomies with humans, for advancements in (pre)clinical applications (Figure 2).

### 3.1 AAV vectors for retinal pigment epithelium

Retinal pigment epithelium (RPE) is a single layer of pigmented cells situated between the retina and the choroid (Figure 1C). RPE plays critical roles in maintaining retinal function and vision, including absorption of scattered light, regeneration of 11-cis-retinal, transportation of nutrients and ions, phagocytosis of shed photoreceptor membranes, and secretion of a variety of growth factors (29). Damage or dysfunction of RPE cells can lead to retinal degenerative diseases, such as age-related macular degeneration, retinitis pigmentosa, and Stargardt’s disease (29) (Figure 1D).

#### 3.1.1 AAV vectors for subretinal injection

Subretinal injection refers to the delivery of drugs into the space between the RPE layer and photoreceptor outer segments. Direct contact of AAV solutions with RPE cells in the confined subretinal space may facilitate transduction.

In basic research, as mentioned earlier, the initial test demonstrated that AAV2/2 could transduce RPE cells and photoreceptors in adult mice via subretinal injection (26). Subsequent studies revealed that other AAV vectors, including AAV2/1, AAV2/4, AAV2/5, AAV2/6, AAV2/7, AAV2/8, and AAV2/9, were also highly effective in targeting RPE cells in rodent retinas, with AAV2/1 and AAV2/4 showing more specific infection of RPE cells (30–33) (Figure 2A).

In clinical practice, Luxturna, the first US FDA-approved gene therapy drug designed to deliver the correct RPE65 gene into RPE cells of patients with biallelic RPE65 mutation, uses AAV2 as a vector through subretinal injection. Apart from AAV2/2, AAV2/4, AAV2/5, and AAV8 have also been employed in clinical trials for delivering genes to RPE cells in patients with several types of inherited retinal diseases (16, 20). Since AAV2/4 was effective and more specific in transducing RPE cells via subretinal injection in a non-human primate model (32), it holds promise as a candidate for (pre)clinical studies when more precise RPE transduction is required (Figure 2A).

#### 3.1.2 AAV vectors for intravitreal injection

While subretinal injection of multiple AAV variants has proven effective in transducing RPE cells, it comes with certain challenges, including surgical requirements, risks of retinal detachment, and limited lateral spreading of AAV solutions. Intravitreal injection, on the other hand, is the delivery of drugs of interest into the vitreous cavity, which is surgically simpler, has no risks of retinal detachment, and enables dispersion throughout the entire retina. However, wild-type AAV vectors administered intravitreally usually do not effectively target RPE cells and photoreceptors on the outer side of the retina. AAV capsid engineering is often necessary to develop AAV variants capable of infecting RPE cells via intravitreal injection.

In basic research, AAV2.7m8, an AAV2 variant created through *in vivo* directed evolution in mice, was able to transduce RPE cells when administered from the vitreous and deliver rescue genes in rd12 mice (34). However, AAV2.7m8 did not achieve the same results in non-human primates (Figure 2A).

In a preclinical study, AAV2.R100, a recently developed AAV2 variant through *in vivo* directed evolution in non-human primates, exhibited decent RPE cell transduction after intravitreal injection (35). AAV2.R100 has been used for gene delivery to RPE cells in clinical trials involving patients with choroideremia (20) (Figure 2A).

#### 3.1.3 AAV vectors for suprachoroidal injection

Suprachoroidal injection is the delivery of drugs into the space between the inner sclera and the outer choroid. Compared with subretinal injection, suprachoroidal injection is less invasive and may facilitate greater spreading.

In basic research, suprachoroidal injection of AAV8 and AAV9 in rats resulted in widespread transgene expression in RPE cells across a large portion of the eye (36). AAV2 and AAV5 have also been reported to transduce RPE cells in rodents after suprachoroidal injection (37) (Figure 2A).

In preclinical studies, AAV8 was capable of transducing a wide range of RPE cells in rhesus monkeys via suprachoroidal injection (36, 38). Suprachoroidal injection of AAV8.anti-VEGFfab (RGX-314) has been used in clinical trials to treat patients with wet age-related macular degeneration and diabetic retinopathy (16, 20), although RGX-314 may target other ocular cells in addition to RPE cells (36, 38) (Figure 2A).

### 3.2 AAV vectors for photoreceptors

Photoreceptors, including rods and cones, are situated at the outer nuclear layer of the retina with their segments in close contact with RPE cells (Figure 1C). Photoreceptors are specialized light-sensing neurons that convert light into neural signals through a sequence of physical and biochemical reactions known as phototransduction. Mutations in a wide diversity of genes involved in the phototransduction cascade could lead to inherited retinal diseases, such as retinitis pigmentosa, Leber congenital amaurosis, and achromatopsia (6) (Figure 1D). Currently, the majority of genes associated with photoreceptor degeneration have been identified (6, 7), providing opportunities to develop relevant gene therapy strategies.

#### 3.2.1 AAV vectors for subretinal injection

In basic research, subretinal injection of AAV2/2 was able to transduce photoreceptors in adult mice, as previously mentioned (26). However, wild-type AAV2 may not be the best vector for photoreceptor gene transfer. AAV2(n)YF, AAV2 variants containing multiple(n) tyrosine-to-phenylalanine mutations on capsid surface, exhibited higher gene delivery efficiency to photoreceptors (39). Other AAV serotypes, including AAV2/5, AAV2/7, AAV2/8, and AAV-DJ, were also highly effective in targeting photoreceptors in rodents after subretinal injection (30, 32, 40, 41) (Figure 2B).

In preclinical studies, AAV2/2, AAV2/5, AAV8, and AAV2tYF have been used for gene delivery to photoreceptors in monkeys (42) and in human clinical trials to treat diseases including retinitis pigmentosa, achromatopsia, and Leber congenital amaurosis (16, 20) (Figure 2B).

#### 3.2.2 AAV vectors for intravitreal injection

In basic research, three AAV2 variants generated through directed evolution, AAV2.7m8, AAV2.GL, and AAV2.NN, could transduce photoreceptors and other retinal cells after intravitreal injection. When these vectors are combined with photoreceptor promoters, photoreceptor-specific gene expression could be achieved (34, 43) (Figure 2B).

In preclinical studies, AAV2.7m8, AAV2.GL, AAV2.NN, and newly identified AAV2.R100, were able to deliver genes into extrafoveal and peripheral photoreceptors in cynomolgus monkeys (34, 43). AAV2.R100 has been used in a clinical trial to transfer the gene encoding retinitis pigmentosa GTPase regulator (RPGR) into photoreceptors to treat X-linked retinitis pigmentosa (20) (Figure 2B).

#### 3.2.3 AAV vectors for suprachoroidal injection

In basic research, suprachoroidal injection of AAV8 and AAV9 has demonstrated the ability to transduce a broad range of photoreceptors in rats (36) (Figure 2B).

In preclinical studies, AAV8 was capable of delivering genes into photoreceptors in addition to RPE cells in rhesus monkeys following suprachoroidal injection (36, 38). As mentioned previously, suprachoroidal delivery of AAV8.anti-VEGFfab (RGX-314), which could infect both photoreceptors and RPE cells, is undergoing clinical trials for the treatment of patients with wet age-related macular degeneration and diabetic retinopathy (16, 20) (Figure 2B).

### 3.3 AAV vectors for retinal ganglion cells

Retinal ganglion cells (RGCs), the sole output neurons of the retina, lie in the innermost cell layer of the retina (Figure 1C). RGC axons form bundles and the nerve fiber layer in the retina and project from the eye to multiple regions of the brain. The inner limiting membrane (ILM), a species-specific variable basement membrane, structurally constitutes the border between the retina and the vitreous (44). Distinct physical, physiological, metabolic, or genetic problems in the eye lead to a variety of RGC degenerative diseases, including glaucoma, traumatic optic neuropathy, and Leber’s hereditary optic neuropathy (45) (Figure 1D).

#### 3.3.1 AAV vectors for intravitreal injection

In basic research, intravitreal injection of AAV2 proved effective in mediating gene transfer in almost all RGCs in adult mice (46). This method has become a commonly used approach to transduce RGCs (47) (Figure 2C).

However, in (pre)clinical studies, the efficiency of RGC transduction by wild-type AAVs through intravitreal injection is dramatically low compared with that in mice. Due to species-specific factors, such as thicker inner limiting membrane in primates, intravitreal injection of wild-type AAV2 in the macaque only targeted a ring of RGCs around the fovea and scattered RGCs in the peripheral retina (48). Although wild-type AAV2 has been used in human clinical trials to treat Leber hereditary optic neuropathy, a mitochondrial disease primarily affecting RGCs (20, 49), its limited efficiency in transducing primates RGCs might be a hindrance to therapeutic efficacy. Intravitreal injection of AAV2.7m8 in cynomolgus monkeys resulted in stronger transgene expression in RGCs, but the transduction regions remained restricted (34). AAV2.7m8 has been employed in human clinical trials to transfer optogenetic genes into RGCs to help the vision of patients with retinitis pigmentosa (16, 20). Intriguingly, AAV2.R100 demonstrated greater effectiveness in targeting RGCs in primates. Intravitreal administration of AAV2.R100 in non-human primates led to widespread and robust transduction of RGCs in both central and peripheral retinal regions (35) (Figure 2C).

#### 3.3.2 AAV vectors for sub-ILM injection

Sub-inner limiting membrane (sub-ILM) injection involves the injection of drugs into the created space between the ILM and the retina in order to circumvent the barrier effect of the primate ILM (50). Although surgically demanding, sub-ILM injection of AAV2 in non-human primates achieved highly efficient RGC transduction in the vicinity of the injection site (51). Similar to subretinal injection, the spread of AAV and consequently the bio-distribution of transgene after sub-ILM are limited by the surgery (Figure 2C).

### 3.4 AAV vectors for Müller glial cells

Müller glial cells, the primary glial cell type in the vertebrate retina, span the entire thickness of the tissue, extending from their basal end feet forming the ILM to their apical microvilli in the subretinal space (Figure 1C). Müller glial cells interact with all retinal neurons and provide them with structural, nutritional, homeostatic, osmotic, metabolic, and growth factor support (52, 53). Some genetic mutations in Müller glial cells, such as CRALBP and CRB1, can lead to retinal degenerative diseases (54, 55). In addition, Müller glial cells have been considered a potential target for retinal therapy to secrete therapeutic factors or to regenerate retinal neurons *in vivo* (53, 56).

#### 3.4.1 AAV vectors for subretinal injection

In basic research, subretinal injection of AAV2/1 and AAV2/9 was able to efficiently transduce Müller glial cells in adult mice (33, 40, 57) (Figure 2D).

In preclinical studies, only a limited number of Müller glial cells were transduced following subretinal injection of AAV2 or AAV8 (42). Obviously, AAV vectors for Müller glial cells via this injection route have not been extensively investigated (Figure 2D).

#### 3.4.2 AAV vectors for intravitreal injection

In basic research, intravitreal injection of AAV6, AAV9, and Anc80 in adult mice has demonstrated the ability to infect a portion of Müller glial cells (52, 57). Notably, AAV6 derivatives, including AAV.ShH10, AAV.ShH10(YF), and AAV.ShH13, targeted Müller glial cells at much higher efficiency and specificity after intravitreal injection (52, 58, 59). AAV.7m8, developed for delivery from the vitreous, also exhibited a strong transducing ability to Müller glial cells in mice (34) (Figure 2D).

In a preclinical study, intravitreal injection of AAV2 was able to infect macaque Müller glial cells but was limited to the perifoveal region (48). Sub-ILM injection in primates may assist AAV2 in transducing Müller glial cells in other regions of the retina. Further studies are required to identify efficient AAV vectors for primate Müller glial cell transduction (Figure 2D).

### 3.5 AAV vectors for ciliary epithelial cells

The ciliary epithelium, located behind the iris and covering the ciliary muscle (Figure 1B), consists of the columnar non-pigmented and the cuboidal pigmented epithelial cells. The non-pigmented epithelial cells are responsible for the secretion of aqueous humor, which is required for the maintenance of proper intraocular pressure and global shape (60). Ciliary epithelial cells have been considered a target of gene therapy to lower intraocular pressure in patients with glaucoma (61–64).

#### 3.5.1 AAV vectors for intracameral injection

Intracameral injection is the delivery of drugs into the anterior chamber of the eye.

In basic research, intracameral injection of self-complementary AAV2 (scAAV2) and scAAV5 could transduce ciliary epithelial cells in both healthy and glaucomatous rodents (65–67). Additionally, AAV-DJ injected intracamerally also exhibited substantial infectivity in the ciliary epithelium in mice (68) (Figure 2E).

In a preclinical study, scAAV2 might target ciliary epithelial cells in cynomolgus monkeys after intracameral injection (67). Further studies are required to identify efficient AAV vectors for primate ciliary epithelial cell transduction (Figure 2E).

#### 3.5.2 AAV vectors for intravitreal injection

In basic research, intravitreal injection of ShH10 efficiently transduced ciliary epithelial cells and lowered intraocular pressure in glaucomatous mice by CRISPR/Cas9-mediated disruption of ciliary body aquaporin 1 (69) (Figure 2E).

In a preclinical study, ShH10 was found to target human *ex vivo* ciliary epithelium from post-mortem donors (69). However, it remains uncertain whether ShH10 can effectively transduce primate ciliary epithelium via intravitreal injection (Figure 2E).

### 3.6 AAV vectors for trabecular meshwork cells

The trabecular meshwork, situated at the iridocorneal angle (Figure 1B), is a spongiform tissue containing endothelial-like cells and extracellular matrices (64). This specialized tissue plays a crucial role in regulating the drainage of aqueous humor from the eye and controlling intraocular pressure (61). Consequently, the trabecular meshwork cells have been considered an ideal target for reducing intraocular pressure in glaucoma gene therapy (61–64).

#### 3.6.1 AAV vectors for intracameral injection

In basic research, scAAV2 and scAAV5 have been widely utilized to target trabecular meshwork cells and lower intraocular pressure in rodents through intracameral injection (65–67, 70). In addition, AAV-DJ and AAV9 injected intracamerally have also demonstrated robust transduction of mouse trabecular meshwork cells (68) (Figure 2F).

In preclinical studies, intracameral injection of scAAV2 could infect trabecular meshwork cells and reduce intraocular pressure by delivering the C3 transferase gene in monkeys (67, 70). Encouragingly, scAAV2 and its capsid variants with single or triple tyrosine-to-phenylalanine mutations exhibited high efficiency in transducing human trabecular meshwork cells in perfused eyes obtained from postmortem human donors (71) (Figure 2F).

#### 3.6.2 AAV vectors for intravitreal injection

Future studies will be necessary to evaluate or engineer AAV vectors targeting trabecular meshwork cells via intravitreal injection (Figure 2F).

### 3.7 AAV vectors for vascular endothelial cells

Vascular endothelial cells, which form the inner layer of blood vessels (Figure 1C), play vital roles in preserving the integrity and functionality of both the choroidal and retinal vascular systems. Damage of choroidal vascular endothelial cells is implicated in the pathogenesis of early atrophic or neovascular age-related macular degeneration (72–74). Similarly, dysfunction of retinal vascular endothelial cells contributes to multiple retinal vascular diseases, including diabetic retinopathy, retinopathy of prematurity, and uveitis (74–76) (Figure 1D). Directly targeting choroidal or retinal vascular endothelial cells holds promise for the treatment of pathological vascular systems in these diseases.

#### 3.7.1 AAV vectors for intravenous injection

For basic research, AAV-BR1, an AAV2 derivative with a peptide inserted into the capsid protein, could target retinal vascular endothelial cells and mediate ectopic gene expression in adult mice after intravenous injection (77, 78). However, it has not been reported whether intravenous injection of AAV-BR1 can effectively transduce choroidal vascular endothelial cells (Figure 2G).

Regarding (pre)clinical studies, AAV vectors targeting choroidal or retinal vascular endothelial cells through intravenous injection have not been thoroughly explored. Nevertheless, it’s worth noting that intravenous injection of AAV will inevitably lead to transduction in cells outside the eye, making it a less than ideal strategy for ocular gene therapy (Figure 2G).

#### 3.7.2 AAV vectors for intravitreal injection

Future studies are required to identify effective AAV vectors to target vascular endothelial cells in the eye through intravitreal injection. It’s important to mention that AAV-BR1 could not transduce mouse retinal vascular endothelial cells after intravitreal injection (78) (Figure 2G).

### 3.8 AAV vectors for pericytes

Pericytes, positioned on the outer surface of capillary blood vessels and separated from underlying endothelial cells by the shared basement membrane (Figure 1C), fulfill diverse functions in vascular homeostasis, including control of blood flow, maintenance of blood-retina/brain barrier, and modulation of angiogenesis (79, 80). Notably, the loss of pericytes and disruption of the blood-retina barrier are implicated in the pathological processes of various ocular vascular diseases, such as diabetic retinopathy, uveitis, and subretinal fibrosis, underscoring the potential for pericytes as targets in gene therapies (79–81) (Figure 1D).

#### 3.8.1 AAV vectors for intravenous injection

AAV-PR, a recently characterized AAV capsid engineered from AAV9, has demonstrated effective transduction of cerebral vascular pericytes and smooth muscle cells after intravenous delivery (82). However, it remains unclear whether AAV-PR can transduce choroidal or retinal vascular pericytes. Future studies are required to identify or engineer AAV vectors targeting choroidal or retinal vascular pericytes through intravenous injection (Figure 2H).

#### 3.8.2 AAV vectors for intravitreal injection

Peri-G, an AAV2/2 derivative with mutations in capsid variable region, was identified in a pioneering study aimed at developing AAV vectors targeting retinal pericytes. Peri-G demonstrated 2.8-fold greater transduction of retinal pericytes than unmodified rAAV2/2 after intravitreal injection (83). Further studies are needed to create AAV vectors with improved targeting efficiency for pericytes in ocular vasculature via intravitreal injection (Figure 2H).

## 4 Perspective

As mentioned above, both basic research and (pre)clinical studies require the development of novel, effective AAV vectors to deliver therapeutic genes into crucial ocular cell types to treat an increasing number of patients with diverse retinal diseases. Encouragingly, more than half a century after the discovery of AAV, we have accumulated a wealth of knowledge about AAV and developed a growing array of strategies to engineer AAV for various purposes (24, 25). Rational design, directed evolution, and bioinformatic reconstruction have proven to be powerful approaches for creating new AAV capsids with unprecedented cell tropisms. For instance, AAV2.R100, a capsid variant able to target multiple primate retinal cell types after intravitreal injection, was identified through *in vivo* directed evolution (35). In addition, optimization of AAV genomic elements, such as promoters, can further increase the cell specificity and efficiency of therapeutic gene expression. Furthermore, engineering AAV vectors to circumvent common challenges associated with AAV-mediated *in vivo* gene delivery, such as limited packaging capacity and host immune response, is equally crucial for retinal gene therapy. In the future, we anticipate the emergence of new AAV vectors that exhibit high efficiency and specificity in targeting distinct ocular cell types, which will tremendously facilitate retinal gene therapy.

## Author contributions

XX: Conceptualization, Visualization, Writing – original draft, Writing – review & editing. XG: Conceptualization, Funding acquisition, Supervision, Writing – original draft, Writing – review & editing.

## References

[1] Barber GW. Physiological chemistry of the eye. Arch Ophthalmol. (1974) 91:141–59. doi: 10.1001/archopht.1974.03900060147013, PMID: 4589813

[2] Bito LZ. The physiology and pathophysiology of intraocular fluids. Exp Eye Res. (1977) 25:273–89. 338321 10.1016/s0014-4835(77)80024-9

[3] Hoon M Okawa H Della Santina L Wong RO. Functional architecture of the retina: development and disease. Prog Retin Eye Res. (2014) 42:44–84. doi: 10.1016/j.preteyeres.2014.06.003, PMID: 24984227 PMC4134977

[4] Campochiaro PA. Molecular pathogenesis of retinal and choroidal vascular diseases. Prog Retin Eye Res. (2015) 49:67–81. doi: 10.1016/j.preteyeres.2015.06.002, PMID: 26113211 PMC4651818

[5] Donegan RK Lieberman RL. Discovery of molecular therapeutics for glaucoma: challenges, successes, and promising directions. J Med Chem. (2016) 59:788–809. doi: 10.1021/acs.jmedchem.5b00828, PMID: 26356532 PMC5547565

[6] Molday RS Moritz OL. Photoreceptors at a glance. J Cell Sci. (2015) 128:4039–45. doi: 10.1242/jcs.175687, PMID: 26574505 PMC4712787

[7] Ben-Yosef T. Inherited retinal diseases. Int J Mol Sci. (2022) 23:13467. doi: 10.3390/ijms232113467 36362249 PMC9654499

[8] Tham YC Li X Wong TY Quigley HA Aung T Cheng CY. Global prevalence of glaucoma and projections of glaucoma burden through 2040: a systematic review and meta-analysis. Ophthalmology. (2014) 121:2081–90. doi: 10.1016/j.ophtha.2014.05.013, PMID: 24974815

[9] Wong WL Su X Li X Cheung CM Klein R Cheng CY. Global prevalence of age-related macular degeneration and disease burden projection for 2020 and 2040: a systematic review and meta-analysis. Lancet Glob Health. (2014) 2:e106–16. doi: 10.1016/S2214-109X(13)70145-1, PMID: 25104651

[10] Teo ZL Tham YC Yu M Chee ML Rim TH Cheung N. Global prevalence of diabetic retinopathy and projection of burden through 2045: systematic review and meta-analysis. Ophthalmology. (2021) 128:1580–91. doi: 10.1016/j.ophtha.2021.04.027, PMID: 33940045

[11] Hanany M Rivolta C Sharon D. Worldwide carrier frequency and genetic prevalence of autosomal recessive inherited retinal diseases. Proc Natl Acad Sci U S A. (2020) 117:2710–6. doi: 10.1073/pnas.1913179117, PMID: 31964843 PMC7007541

[12] Hedengran A Kolko M. The molecular aspect of anti-glaucomatous eye drops—are we harming our patients? Mol Asp Med. (2023) 93:101195. doi: 10.1016/j.mam.2023.101195, PMID: 37459821

[13] Vorwerk C Thelen U Buchholz P Kimmich F. Treatment of glaucoma patients with insufficient intraocular pressure control: a survey of German ophthalmologists in private practice. Curr Med Res Opin. (2008) 24:1295–301. doi: 10.1185/030079908X291976, PMID: 18366862

[14] Malihi M Moura Filho ER Hodge DO Sit AJ. Long-term trends in glaucoma-related blindness in Olmsted County, Minnesota. Ophthalmology. (2014) 121:134–41. doi: 10.1016/j.ophtha.2013.09.003, PMID: 24823760 PMC4038428

[15] Liu MM Tuo J Chan CC. Gene therapy for ocular diseases. Br J Ophthalmol. (2011) 95:604–12. doi: 10.1136/bjo.2009.174912, PMID: 20733027 PMC3154727

[16] Ghoraba HH Akhavanrezayat A Karaca I Yavari N Lajevardi S Hwang J. Ocular gene therapy: a literature review with special focus on immune and inflammatory responses. Clin Ophthalmol. (2022) 16:1753–71. doi: 10.2147/OPTH.S364200 35685379 PMC9173725

[17] FDA approves hereditary blindness gene therapy. Nat Biotechnol. (2018) 36:6. doi: 10.1038/nbt0118-6a, PMID: 29319697

[18] Kahraman NS Oner A Ozkul Y Dundar M. Frequency of RPE65 gene mutation in patients with hereditary retinal dystrophy. Turk J Ophthalmol. (2022) 52:270–5. doi: 10.4274/tjo.galenos.2021.74944, PMID: 36017377 PMC9421938

[19] Wasnik VB Thool AR. Ocular gene therapy: a literature review with focus on current clinical trials. Cureus. (2022) 14:e29533. doi: 10.7759/cureus.29533, PMID: 36312652 PMC9590687

[20] Ail D Malki H Zin EA Dalkara D. Adeno-associated virus (AAV)—based gene therapies for retinal diseases: where are we? Appl Clin Genet. (2023) 16:111–30. doi: 10.2147/TACG.S383453 37274131 PMC10239239

[21] Arabi F Mansouri V Ahmadbeigi N. Gene therapy clinical trials, where do we go? An overview. Biomed Pharmacother. (2022) 153:113324. doi: 10.1016/j.biopha.2022.113324, PMID: 35779421

[22] Samulski RJ Muzyczka N. AAV-mediated gene therapy for research and therapeutic purposes. Annu Rev Virol. (2014) 1:427–51. doi: 10.1146/annurev-virology-031413-085355, PMID: 26958729

[23] Brommel CM Cooney AL Sinn PL. Adeno-associated virus-based gene therapy for lifelong correction of genetic disease. Hum Gene Ther. (2020) 31:985–95. doi: 10.1089/hum.2020.138, PMID: 32718227 PMC7495917

[24] Li C Samulski RJ. Engineering adeno-associated virus vectors for gene therapy. Nat Rev Genet. (2020) 21:255–72. doi: 10.1038/s41576-019-0205-4, PMID: 32042148

[25] Wang D Tai PWL Gao G. Adeno-associated virus vector as a platform for gene therapy delivery. Nat Rev Drug Discov. (2019) 18:358–78. doi: 10.1038/s41573-019-0012-9, PMID: 30710128 PMC6927556

[26] Ali RR Reichel MB Thrasher AJ Levinsky RJ Kinnon C Kanuga N. Gene transfer into the mouse retina mediated by an adeno-associated viral vector. Hum Mol Genet. (1996) 5:591–4. doi: 10.1093/hmg/5.5.591, PMID: 8733124

[27] Trapani I Puppo A Auricchio A. Vector platforms for gene therapy of inherited retinopathies. Prog Retin Eye Res. (2014) 43:108–28. doi: 10.1016/j.preteyeres.2014.08.001, PMID: 25124745 PMC4241499

[28] Tian B Bilsbury E Doherty S Teebagy S Wood E Su W. Ocular drug delivery: advancements and innovations. Pharmaceutics. (2022) 14:1931. doi: 10.3390/pharmaceutics14091931, PMID: 36145679 PMC9506479

[29] Strauss O. The retinal pigment epithelium in visual function. Physiol Rev. (2005) 85:845–81. doi: 10.1152/physrev.00021.2004, PMID: 15987797

[30] Lebherz C Maguire A Tang W Bennett J Wilson JM. Novel AAV serotypes for improved ocular gene transfer. J Gene Med. (2008) 10:375–82. doi: 10.1002/jgm.1126, PMID: 18278824 PMC2842078

[31] Yang GS Schmidt M Yan Z Lindbloom JD Harding TC Donahue BA. Virus-mediated transduction of murine retina with adeno-associated virus: effects of viral capsid and genome size. J Virol. (2002) 76:7651–60. doi: 10.1128/JVI.76.15.7651-7660.2002, PMID: 12097579 PMC136354

[32] Weber M Rabinowitz J Provost N Conrath H Folliot S Briot D. Recombinant adeno-associated virus serotype 4 mediates unique and exclusive long-term transduction of retinal pigmented epithelium in rat, dog, and nonhuman primate after subretinal delivery. Mol Ther. (2003) 7:774–81. doi: 10.1016/S1525-0016(03)00098-4, PMID: 12788651

[33] Auricchio A Kobinger G Anand V Hildinger M O’Connor E Maguire AM. Exchange of surface proteins impacts on viral vector cellular specificity and transduction characteristics: the retina as a model. Hum Mol Genet. (2001) 10:3075–81. doi: 10.1093/hmg/10.26.3075, PMID: 11751689

[34] Dalkara D Byrne LC Klimczak RR Visel M Yin L Merigan WH. *In vivo*-directed evolution of a new adeno-associated virus for therapeutic outer retinal gene delivery from the vitreous. Sci Transl Med. (2013) 5:189ra76. doi: 10.1126/scitranslmed.3005708 23761039

[35] Kotterman M Beliakoff G Croze R Vazin T Schmitt C Szymanski P. Directed evolution of AAV targeting primate retina by intravitreal injection identifies R100, a variant demonstrating robust gene delivery and therapeutic efficacy in non-human primates. *bioRxiv*. Available at: 10.1101/2021.06.24.449775. [Epub ahead of preprint]

[36] Ding K Shen J Hafiz Z Hackett SF Silva RLE Khan M. AAV8-vectored suprachoroidal gene transfer produces widespread ocular transgene expression. J Clin Invest. (2019) 129:4901–11. doi: 10.1172/JCI129085, PMID: 31408444 PMC6819121

[37] Kansara V Muya L Wan CR Ciulla TA. Suprachoroidal delivery of viral and nonviral gene therapy for retinal diseases. J Ocul Pharmacol Ther. (2020) 36:384–92. doi: 10.1089/jop.2019.0126, PMID: 32255727 PMC7404827

[38] Yiu G Chung SH Mollhoff IN Nguyen UT Thomasy SM Yoo J. Suprachoroidal and subretinal injections of AAV using transscleral microneedles for retinal gene delivery in nonhuman primates. Mol Ther Methods Clin Dev. (2020) 16:179–91. doi: 10.1016/j.omtm.2020.01.002, PMID: 32055646 PMC7005511

[39] Petrs-Silva H Dinculescu A Li Q Deng WT Pang JJ Min SH. Novel properties of tyrosine-mutant AAV2 vectors in the mouse retina. Mol Ther. (2011) 19:293–301. doi: 10.1038/mt.2010.234, PMID: 21045809 PMC3034844

[40] Allocca M Mussolino C Garcia-Hoyos M Sanges D Iodice C Petrillo M. Novel adeno-associated virus serotypes efficiently transduce murine photoreceptors. J Virol. (2007) 81:11372–80. doi: 10.1128/JVI.01327-07, PMID: 17699581 PMC2045569

[41] Katada Y Kobayashi K Tsubota K Kurihara T. Evaluation of AAV-DJ vector for retinal gene therapy. PeerJ. (2019) 7:e6317. doi: 10.7717/peerj.6317, PMID: 30671314 PMC6339780

[42] Vandenberghe LH Bell P Maguire AM Cearley CN Xiao R Calcedo R. Dosage thresholds for AAV2 and AAV8 photoreceptor gene therapy in monkey. Sci Transl Med. (2011) 3:88ra54. doi: 10.1126/scitranslmed.3002103 PMC5027886 21697530

[43] Pavlou M Schon C Occelli LM Rossi A Meumann N Boyd RF. Novel AAV capsids for intravitreal gene therapy of photoreceptor disorders. EMBO Mol Med. (2021) 13:e13392. doi: 10.15252/emmm.202013392, PMID: 33616280 PMC8033523

[44] Peynshaert K Devoldere J Minnaert AK De Smedt SC Remaut K. Morphology and composition of the inner limiting membrane: species-specific variations and relevance toward drug delivery research. Curr Eye Res. (2019) 44:465–75. doi: 10.1080/02713683.2019.1565890, PMID: 30638413

[45] Levin LA Gordon LK. Retinal ganglion cell disorders: types and treatments. Prog Retin Eye Res. (2002) 21:465–84. doi: 10.1016/S1350-9462(02)00012-5, PMID: 12207946

[46] Park KK Liu K Hu Y Smith PD Wang C Cai B. Promoting axon regeneration in the adult CNS by modulation of the PTEN/mTOR pathway. Science. (2008) 322:963–6. doi: 10.1126/science.1161566, PMID: 18988856 PMC2652400

[47] Guo X Starr C Zhou J Chen B. Protocol for evaluating the role of a gene in protecting mouse retinal ganglion cells. STAR Protoc. (2021) 2:100932. doi: 10.1016/j.xpro.2021.100932, PMID: 34806045 PMC8581646

[48] Yin L Greenberg K Hunter JJ Dalkara D Kolstad KD Masella BD. Intravitreal injection of AAV2 transduces macaque inner retina. Invest Ophthalmol Vis Sci. (2011) 52:2775–83. doi: 10.1167/iovs.10-6250, PMID: 21310920 PMC3088562

[49] Sahel JA Newman NJ Yu-Wai-Man P Vignal-Clermont C Carelli V Biousse V. Gene therapies for the treatment of Leber hereditary optic neuropathy. Int Ophthalmol Clin. (2021) 61:195–208. doi: 10.1097/IIO.0000000000000364, PMID: 34584057 PMC8478322

[50] Gamlin PD Alexander JJ Boye SL Witherspoon CD Boye SE. SubILM injection of AAV for gene delivery to the retina. Methods Mol Biol. (2019) 1950:249–62. doi: 10.1007/978-1-4939-9139-6_14 30783978 PMC6700748

[51] Boye SE Alexander JJ Witherspoon CD Boye SL Peterson JJ Clark ME. Highly efficient delivery of adeno-associated viral vectors to the primate retina. Hum Gene Ther. (2016) 27:580–97. doi: 10.1089/hum.2016.085, PMID: 27439313 PMC4991591

[52] Pellissier LP Hoek RM Vos RM Aartsen WM Klimczak RR Hoyng SA. Specific tools for targeting and expression in Müller glial cells. Mol Ther Methods Clin Dev. (2014) 1:14009. doi: 10.1038/mtm.2014.9, PMID: 26015954 PMC4362388

[53] Bringmann A Pannicke T Grosche J Francke M Wiedemann P Skatchkov SN. Müller cells in the healthy and diseased retina. Prog Retin Eye Res. (2006) 25:397–424. doi: 10.1016/j.preteyeres.2006.05.003, PMID: 16839797

[54] Maw MA Kennedy B Knight A Bridges R Roth KE Mani EJ. Mutation of the gene encoding cellular retinaldehyde-binding protein in autosomal recessive retinitis pigmentosa. Nat Genet. (1997) 17:198–200. doi: 10.1038/ng1097-198, PMID: 9326942

[55] den Hollander AI ten Brink JB de Kok YJ van Soest S van den Born LI van Driel MA. Mutations in a human homologue of *Drosophila* crumbs cause retinitis pigmentosa (RP12). Nat Genet. (1999) 23:217–21. doi: 10.1038/13848 10508521

[56] Devoldere J Peynshaert K De Smedt SC Remaut K. Müller cells as a target for retinal therapy. Drug Discov Today. (2019) 24:1483–98. doi: 10.1016/j.drudis.2019.01.023, PMID: 30731239

[57] Schwartz MK Likhite S Vetter TA Baird MC McGovern V Sierra Delgado A. In-depth comparison of Anc80L65 and AAV9 retinal targeting and characterization of cross-reactivity to multiple AAV serotypes in humans. Mol Ther Methods Clin Dev. (2023) 30:16–29. doi: 10.1016/j.omtm.2023.05.016, PMID: 37746244 PMC10512013

[58] Klimczak RR Koerber JT Dalkara D Flannery JG Schaffer DV. A novel adeno-associated viral variant for efficient and selective intravitreal transduction of rat Müller cells. PLoS One. (2009) 4:e7467. doi: 10.1371/journal.pone.0007467, PMID: 19826483 PMC2758586

[59] Koerber JT Klimczak R Jang JH Dalkara D Flannery JG Schaffer DV. Molecular evolution of adeno-associated virus for enhanced glial gene delivery. Mol Ther. (2009) 17:2088–95. doi: 10.1038/mt.2009.184, PMID: 19672246 PMC2788045

[60] Goel M Picciani RG Lee RK Bhattacharya SK. Aqueous humor dynamics: a review. Open Ophthalmol J. (2010) 4:52–9. doi: 10.2174/1874364101004010052, PMID: 21293732 PMC3032230

[61] Demetriades AM. Gene therapy for glaucoma. Curr Opin Ophthalmol. (2011) 22:73–7. doi: 10.1097/ICU.0b013e32834371d2, PMID: 21252673

[62] Liu X Rasmussen CA Gabelt BT Brandt CR Kaufman PL. Gene therapy targeting glaucoma: where are we? Surv Ophthalmol. (2009) 54:472–86. doi: 10.1016/j.survophthal.2009.04.003, PMID: 19539835 PMC2848072

[63] Hakim A Guido B Narsineni L Chen DW Foldvari M. Gene therapy strategies for glaucoma from IOP reduction to retinal neuroprotection: Progress towards non-viral systems. Adv Drug Deliv Rev. (2023) 196:114781. doi: 10.1016/j.addr.2023.114781, PMID: 36940751

[64] Borras T Brandt CR Nickells R Ritch R. Gene therapy for glaucoma: treating a multifaceted, chronic disease. Invest Ophthalmol Vis Sci. (2002) 43:2513–8. PMID: 12147578

[65] Bogner B Boye SL Min SH Peterson JJ Ruan Q Zhang Z. Capsid mutated adeno-associated virus delivered to the anterior chamber results in efficient transduction of trabecular meshwork in mouse and rat. PLoS One. (2015) 10:e0128759. doi: 10.1371/journal.pone.0128759, PMID: 26052939 PMC4460001

[66] Lee SH Sim KS Kim CY Park TK. Transduction pattern of AAVs in the trabecular meshwork and anterior-segment structures in a rat model of ocular hypertension. Mol Ther Methods Clin Dev. (2019) 14:197–205. doi: 10.1016/j.omtm.2019.06.009, PMID: 31406700 PMC6685643

[67] Buie LK Rasmussen CA Porterfield EC Ramgolam VS Choi VW Markovic-Plese S. Self-complementary AAV virus (scAAV) safe and long-term gene transfer in the trabecular meshwork of living rats and monkeys. Invest Ophthalmol Vis Sci. (2010) 51:236–48. doi: 10.1167/iovs.09-3847, PMID: 19684004 PMC2869048

[68] Qiao Y Sun Z Tan C Lai J Sun X Chen J. Intracameral injection of AAV-DJ.COMP-ANG1 reduces the IOP of mice by reshaping the trabecular outflow pathway. Invest Ophthalmol Vis Sci. (2022) 63:15. doi: 10.1167/iovs.63.13.15 PMC9769031 36520455

[69] Wu J Bell OH Copland DA Young A Pooley JR Maswood R. Gene therapy for glaucoma by ciliary body aquaporin 1 disruption using CRISPR-Cas9. Mol Ther. (2020) 28:820–9. doi: 10.1016/j.ymthe.2019.12.012, PMID: 31981492 PMC7054720

[70] Tan J Wang X Cai S He F Zhang D Li D. C3 transferase-expressing scAAV2 transduces ocular anterior segment tissues and lowers intraocular pressure in mouse and monkey. Mol Ther Methods Clin Dev. (2020) 17:143–55. doi: 10.1016/j.omtm.2019.11.017, PMID: 31909087 PMC6938898

[71] Rodriguez-Estevez L Asokan P Borras T. Transduction optimization of AAV vectors for human gene therapy of glaucoma and their reversed cell entry characteristics. Gene Ther. (2020) 27:127–42. doi: 10.1038/s41434-019-0105-4, PMID: 31611639 PMC7153980

[72] Voigt AP Mullin NK Mulfaul K Lozano LP Wiley LA Flamme-Wiese MJ. Choroidal endothelial and macrophage gene expression in atrophic and neovascular macular degeneration. Hum Mol Genet. (2022) 31:2406–23. doi: 10.1093/hmg/ddac043, PMID: 35181781 PMC9307320

[73] Yeo NJY Chan EJJ Cheung C. Choroidal neovascularization: mechanisms of endothelial dysfunction. Front Pharmacol. (2019) 10:1363. doi: 10.3389/fphar.2019.01363 31849644 PMC6895252

[74] Alizadeh E Mammadzada P Andre H. The different facades of retinal and choroidal endothelial cells in response to hypoxia. Int J Mol Sci. (2018) 19:3846. doi: 10.3390/ijms19123846, PMID: 30513885 PMC6321100

[75] Bharadwaj AS Appukuttan B Wilmarth PA Pan Y Stempel AJ Chipps TJ. Role of the retinal vascular endothelial cell in ocular disease. Prog Retin Eye Res. (2013) 32:102–80. doi: 10.1016/j.preteyeres.2012.08.004, PMID: 22982179 PMC3679193

[76] Mrugacz M Bryl A Zorena K. Retinal vascular endothelial cell dysfunction and neuroretinal degeneration in diabetic patients. J Clin Med. (2021) 10. doi: 10.3390/jcm10030458, PMID: 33504108 PMC7866162

[77] Korbelin J Dogbevia G Michelfelder S Ridder DA Hunger A Wenzel J. A brain microvasculature endothelial cell-specific viral vector with the potential to treat neurovascular and neurological diseases. EMBO Mol Med. (2016) 8:609–25. doi: 10.15252/emmm.201506078, PMID: 27137490 PMC4888852

[78] Ivanova E Corona C Eleftheriou CG Stout RF Jr Korbelin J Sagdullaev BT. AAV-BR1 targets endothelial cells in the retina to reveal their morphological diversity and to deliver Cx43. J Comp Neurol. (2022) 530:1302–17. doi: 10.1002/cne.25277, PMID: 34811744 PMC8969189

[79] Trost A Lange S Schroedl F Bruckner D Motloch KA Bogner B. Brain and retinal pericytes: origin, function and role. Front Cell Neurosci. (2016) 10:20. doi: 10.3389/fncel.2016.00020, PMID: 26869887 PMC4740376

[80] Caporarello N D’Angeli F Cambria MT Candido S Giallongo C Salmeri M. Pericytes in microvessels: from “mural” function to brain and retina regeneration. Int J Mol Sci. (2019) 20:6351. doi: 10.3390/ijms20246351, PMID: 31861092 PMC6940987

[81] Luo X Yang S Liang J Zhai Y Shen M Sun J. Choroidal pericytes promote subretinal fibrosis after experimental photocoagulation. Dis Model Mech. (2018) 11:dmm032060. doi: 10.1242/dmm.032060, PMID: 29622551 PMC5963858

[82] Ramirez SH Hale JF McCarthy S Cardenas CL Dona K Hanlon KS. An engineered adeno-associated virus capsid mediates efficient transduction of pericytes and smooth muscle cells of the brain vasculature. Hum Gene Ther. (2023) 34:682–96. doi: 10.1089/hum.2022.211, PMID: 37376759 PMC10457656

[83] Patel DD Marsic D Periasamy R Zolotukhin S Lipinski DM. Identification of novel retinal pericyte-targeting rAAV vectors through directed evolution. Transl Vis Sci Technol. (2022) 11:28. doi: 10.1167/tvst.11.8.28, PMID: 36018583 PMC9428359

